# Supplementary material for: Atomically dispersed golds on degradable zero-valent copper nanocubes augment oxygen driven Fenton-like reaction for effective orthotopic tumor therapy
Source: Nat Commun. 2022 Dec 15;13:7772. doi: 10.1038/s41467-022-35515-8 (PMC9755215; doi:10.1038/s41467-022-35515-8)
Supplement: Supplementary file 1 — Supplementary infromation [file 41467_2022_35515_MOESM1_ESM.pdf]

Supplementary information

Atomically dispersed golds on degradable zero-valent copper nanocubes augment oxygen driven Fenton-like reaction for effective orthotopic tumor therapy

Liu-Chun Wang[1], Li-Chan Chang[2], Wen-Qi Chen[1], Yi-Hsin Chien[3], Po-Ya Chang[4], Chih-Wen Pao[4], Yin-Fen Liu[2], Hwo-Shuenn Sheu[4,*], Wen-Pin Su[2,5,*], Chen-Hao Yeh[3,*], Chen-Sheng Yeh[1,*]

[1]Department of Chemistry, National Cheng Kung University, Tainan 701, Taiwan

[2]Institute of Clinical Medicine, College of Medicine, National Cheng Kung University, Tainan 704, Taiwan

[3]Department of Materials Science and Engineering, Feng Chia University, Taichung, 40724, Taiwan

[4]National Synchrotron Radiation Research Center, Hsinchu 30076, Taiwan.

[5]Departments of Oncology and Internal Medicine, National Cheng Kung University Hospital, College of Medicine, National Cheng Kung University, Tainan 704, Taiwan

* Corresponding author e-mail: hsheu@nsrrc.org.tw, wpsu@mail.ncku.edu.tw, chenhyeh@fcu.edu.tw, csyeh@mail.ncku.edu.tw

Supplementary Table 1. Grain sizes of Au/Cu^0 nanocubes

sample	phase	Grain size (nm)	phase	Grain size (nm)
$Au_{0.02}Cu_{0.98}$	Au	2.55	Cu	39.11
$Au_{0.05}Cu_{0.95}$	Au	3.09	Cu	35.03
$Au_{0.1}Cu_{0.9}$	Au	3.57	Cu	38.31
$Au_{0.5}Cu_{0.5}$	Au	3.87	Cu	33.81
Cu NP			Cu	35.40

Supplementary Table 2. Rietveld refinement parameters of Au/Cu^0 nanocubes

Sample	Phase	Space group	Unit cell a (Å)	Rwp	Rp	wt %	atomic ratio %
$Au_{0.02}Cu_{0.98}$	Cu	Fm-3m	3.61210(2)	0.0461	0.0288	83.5(7)	94.0(4)
	Au	Fm-3m	3.980(3)			16.5(2)	5.99(8)
$Au_{0.02}Cu_{0.98}$_SA	Cu	Fm-3m	3.62029(3)	0.0453	0.0220	83.8(4)	94.6(3)
	Au1	Fm-3m	4.032(5)			14.7(4)	4.82(2)
	Au2	Fm-3m	3.961(3)			1.47(2)	0.53(7)
$Au_{0.05}Cu_{0.95}$	Cu	Fm-3m	3.61425(3)	0.0430	0.0278	76.5(1)	91.0(4)
	Au	Fm-3m	4.002(1)			23.5(2)	9.0(8)
$Au_{0.1}Cu_{0.9}$	Cu	Fm-3m	3.61875(3)	0.0366	0.0207	62.3(2)	83.7(6)
	Au1	Fm-3m	3.967(2)			9.98(4)	4.3(2)
	Au2	Fm-3m	4.069(1)			27.7(4)	12.0(2)
$Au_{0.5}Cu_{0.5}$	Cu	Fm-3m	3.62537(8)	0.0497	0.0295	37.6(3)	65.1(2)
	Au	Fm-3m	4.015(1)			62.4(2)	34.9(7)
Cu NP	Cu	Fm-3m	3.61569(3)	0.0633	0.0256	100	100

wt %: weight percentage, atomic ratio %: element molar percentage

Supplementary Table 3. Fitting parameters of EXAFS for Au/Cu^0 nanocubes

sample	Center atom	K range	R range	S_0^2	R factor	E_0	$N_{variable}$
$Au_{0.02}Cu_{0.98}$	Au	3.0 – 10.5	1.65 - 3.27	0.9	0.016	6.675	7
$Au_{0.05}Cu_{0.95}$	Au	3.0 - 12.0	1.16 - 3.21	0.9	0.008	4.172	7
$Au_{0.1}Cu_{0.9}$	Au	3.0 – 11.5	1.6 – 3.28	0.9	0.003	5.579	7
$Au_{0.5}Cu_{0.5}$	Au	3.0 - 12	1.7 - 3.17	0.9	0.006	3.921	7
Au foil	Au	2.9 - 12.7	1.68 - 3.3	0.852	0.002	4.878	4
$Au_{0.02}Cu_{0.98}$	Cu	3.0 - 13.6	1.5 - 2.9	0.9	0.003	4.086	4
$Au_{0.05}Cu_{0.95}$	Cu	3.0 - 12.46	1.4 - 2.8	0.9	0.009	3.196	4
$Au_{0.1}Cu_{0.9}$	Cu	3.0 – 12.5	1.7 – 2.8	0.9	0.021	2.392	4
$Au_{0.5}Cu_{0.5}$	Cu	3.0 - 12.5	1.7 - 2.8	0.9	0.017	2.850	4
Cu NP	Cu	2.5 - 12	1.7 – 2.8	0.9	0.005	4.439	4
Cu foil	Cu	2.5 - 13.6	1 - 3	0.894	0.014	4.834	4

Supplementary Table 4. Calculated reaction barriers (Ea in eV) and reaction energies (ΔE in eV) for elementary reactions of H_2O dehydrogenation to H atoms on the Au/Cu^0 nanocubes. The values outside and inside the parentheses represent with and without considering the solvent effect.

**Elementary steps**	$\mathbf{E_a}$	$\mathbf{\Delta E}$
Cu(100)		
$H_2O_{(a)} \rightarrow H_{(a)} + OH_{(a)}$	1.48 (1.38)	0.10 (-0.06)
$H_{(a)} + OH_{(a)} \rightarrow 2H_{(a)} + O_{(a)}$	1.59 (1.57)	0.52 (0.47)
$Au_{0.02}Cu_{0.98}$		
$H_2O_{(a)} \rightarrow H_{(a)} + OH_{(a)}$	1.49 (1.41)	0.17 (0.03)
$H_{(a)} + OH_{(a)} \rightarrow 2H_{(a)} + O_{(a)}$	1.62 (1.61)	0.54 (0.50)
$Au_{0.5}Cu_{0.5}$		
$H_2O_{(a)} \rightarrow H_{(a)} + OH_{(a)}$	1.44 (1.39)	0.83 (0.73)
$H_{(a)} + OH_{(a)} \rightarrow 2H_{(a)} + O_{(a)}$	1.99 (1.99)	1.18 (1.12)
Au(100)		
$H_2O_{(a)} \rightarrow H_{(a)} + OH_{(a)}$	1.88 (1.85)	1.05 (0.93)
$H_{(a)} + OH_{(a)} \rightarrow 2H_{(a)} + O_{(a)}$	2.15 (2.15)	1.93 (1.83)

Supplementary Table 5. The calculated adsorption energy (E_{ads}) of the adsorption of H_2O molecule on all the possible adsorption sites at Cu(100), $Au_{0.02}Cu_{0.98}$(100), $Au_{0.5}Cu_{0.5}$(100), and Au(100) surfaces. The adsorption energy values outside and inside the parentheses represent with and without considering the solvent effect.

Surface	Site	E_{ads} (eV)	Surface	Site	E_{ads} (eV)
Cu	T	-0.36 (-0.41)	Au	T	-0.22 (-0.32)
	T_1	-0.39 (-0.46)		T_1	-0.40 (-0.51)
$Au_{0.02}Cu_{0.98}$	T_2	-0.35 (-0.41)	$Au_{0.5}Cu_{0.5}$	T_2	-0.20 (-0.28)
	T_3	-0.15 (-0.23)			

Supplementary Table 6. The calculated adsorption energy (E_{ads}) of the adsorption of OH group on all the possible adsorption sites at Cu(100), $Au_{0.02}Cu_{0.98}$(100), $Au_{0.5}Cu_{0.5}$(100), and Au(100) surfaces. The adsorption energy values outside and inside the parentheses represent with and without considering the solvent effect.

Surface	Site	E_{ads} (eV)	Surface	Site	E_{ads} (eV)
Cu	T(→H)[a]	-3.46 (-3.54)	Au	T(→B)[a]	-2.59 (-2.67)
	H	-3.46 (-3.54)		H(→B)[a]	-2.59 (-2.67)
	B	-3.42 (-3.50)		B	-2.59 (-2.67)
$Au_{0.02}Cu_{0.98}$	T_1(→H_1)[a]	-3.42 (-3.51)	$Au_{0.5}Cu_{0.5}$	T_1	-2.33 (-2.40)
	T_2(→H_1)[a]	-3.42 (-3.51)		T_2	-1.65 (-1.68)
	T_3(→B_2)[a]	-2.97 (-3.03)		B	-2.94 (-3.03)
	B_1	-3.37 (-3.45)		H_1	-2.85 (-2.95)
	B_2	-2.97 (-3.03)		H_2	-2.85 (-2.94)
	H_1	-3.42 (-3.51)			
	H_2	-3.18 (-3.28)			

[a] The left and right parts of the arrow show that the adsorbate is adsorbed on the original active sites before optimization and on the final active sites after optimization, respectively.

Supplementary Table 7. The calculated adsorption energy (E_{ads}) of the adsorption of O atom on all the possible adsorption sites at Cu(100), $Au_{0.02}Cu_{0.98}$(100), $Au_{0.5}Cu_{0.5}$(100), and Au(100) surfaces. The adsorption energy values outside and inside the parentheses represent with and without considering the solvent effect.

Surface	Site	E_{ads} (eV)	Surface	Site	E_{ads} (eV)
Cu	T(→H)[a]	-5.58 (-5.51)	Au	T(→B)[a]	-3.65 (-3.51)
	H	-5.58 (-5.51)		H	-3.54 (-3.45)
	B(→H)[a]	-5.58 (-5.51)		B	-3.65 (-3.51)
$Au_{0.02}Cu_{0.98}$	T_1(→H_1)[a]	-5.53 (-5.46)	$Au_{0.5}Cu_{0.5}$	T_1	-3.38 (-3.10)
	T_2(→H_1)[a]	-5.53 (-5.46)		T_2	-3.00 (-2.69)
	T_3(→H_2)[a]	-5.09 (-5.00)		B	-4.20 (-4.06)
	B_1(→H_2)[a]	-5.09 (-5.00)		H_1	-4.31 (-4.22)
	B_2(→H_2)[a]	-5.09 (-5.00)		H_2	-4.58 (-4.51)
	H_1	-5.53 (-5.46)			
	H_2	-5.09 (-5.00)			

[a] The left and right parts of the arrow show that the adsorbate is adsorbed on the original active sites before optimization and on the final active sites after optimization, respectively.

Supplementary Table 8. The calculated adsorption energy (E_{ads}) of the adsorption of H atom on all the possible adsorption sites at Cu(100), $Au_{0.02}Cu_{0.98}$(100), $Au_{0.5}Cu_{0.5}$(100), and Au(100) surfaces. The adsorption energy values outside and inside the parentheses represent with and without considering the solvent effect.

Surface	Site	E_{ads} (eV)	Surface	Site	E_{ads} (eV)
Cu	T(→H)[a]	-3.62 (-3.61)	Au	T	-3.10 (-3.08)
	H	-3.62 (-3.61)		H(→B [a]	-3.39 (-3.37)
	B	-3.51 (-3.49)		B	-3.39 (-3.37)
$Au_{0.02}Cu_{0.98}$	T_1(→H_1)[a]	-3.62 (-3.61)	$Au_{0.5}Cu_{0.5}$	T_1(→H_1)[a]	-3.43 (-3.41)
	T_2(→H_2)[a]	-3.47 (-3.46)		T_2	-3.21 (-3.19)
	T_3	-3.12 (-3.08)		B	-3.39 (-3.38)
	B_1	-3.48 (-3.46)		H_1	-3.43 (-3.41)
	B_2	-3.40 (-3.38)		H_2(→B)[a]	-3.39 (-3.38)
	H_1	-3.62 (-3.61)			
	H_2	-3.47 (-3.46)			

[a] The left and right parts of the arrow show that the adsorbate is adsorbed on the original active sites before optimization and on the final active sites after optimization, respectively.

Supplementary Table 9. The calculated adsorption energy (E_{ads}) of the adsorption of O_2 molecule by side-on and end-on configurations on all the possible adsorption sites at Cu(100), $Au_{0.02}Cu_{0.98}$(100), $Au_{0.5}Cu_{0.5}$(100), and Au(100) surfaces. The adsorption energy values outside and inside the parentheses represent with and without considering the solvent effect.

Surface	Configuration	Site	E_{ads} (eV)	Surface	Configuration	Site	E_{ads} (eV)
Cu(100)	Side-on	H	-2.12 (-1.96)	Au(100)	Side-on	H	-0.42 (-0.33)
	End-on	H	-0.86 (-0.62)		End-on	T	-0.21 (-0.17)
$Au_{0.02}Cu_{0.98}$(100)	Side-on	H_1	-2.00 (-1.84)	$Au_{0.5}Cu_{0.5}$(100)	Side-on	H_1	-1.16 (-1.03)
	Side-on	H_2	-1.60 (-1.44)		Side-on	H_2	-1.15 (-1.02)
	End-on	H_1	-0.81 (-0.55)		End-on	H_1	-0.24 (-0.04)
	End-on	H_2	-0.62 (-0.37)		End-on	$H_2(\rightarrow T_1)$[a]	-0.28 (-0.10)
	End-on	T_3	n. s.		End-on	T_2	n. s.

[a] The left and right parts of the arrow show that the adsorbate is adsorbed on the original active sites before optimization and on the final active sites after optimization, respectively.
n. s.: not stable (the adsorption energy is positive)

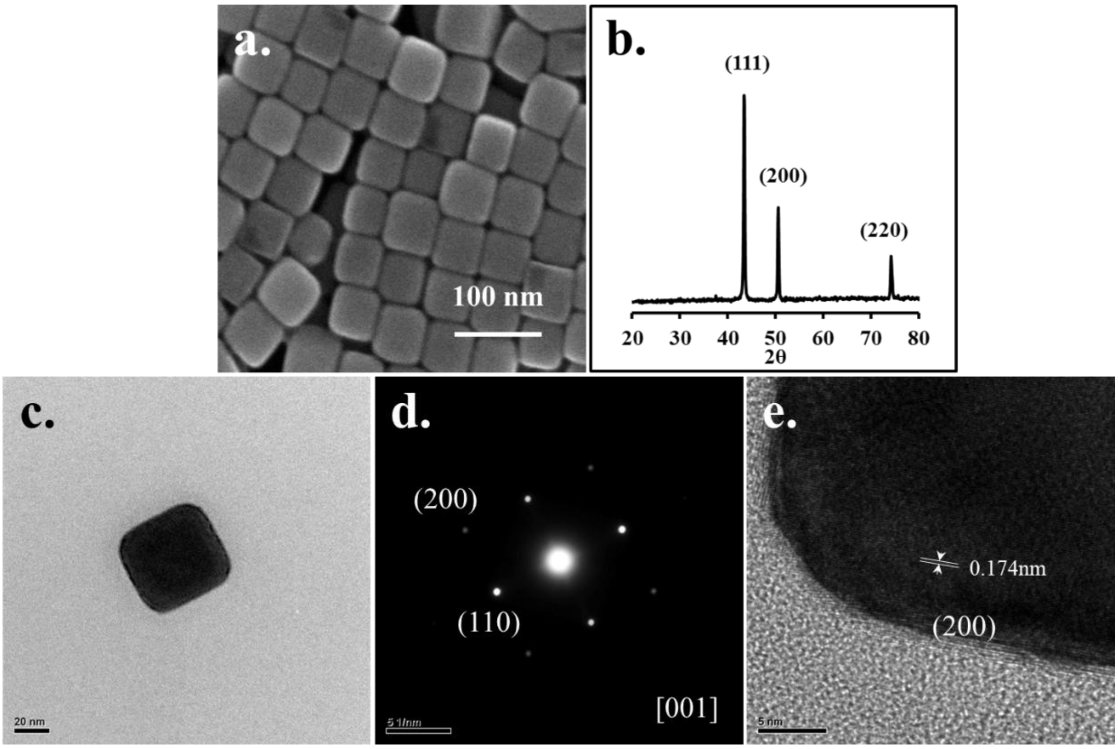

Supplementary Fig. 1. The characteristics of Cu nanocubes. **a.** Scanning electron micrograph (SEM) image of Cu nanocubes. **b.** XRD results indicating a Cu signal (JCPDS cards 03-1018) with a face-centered cubic crystal structure **c-e.** High-resolution TEM image of the single Cu nanocube and the corresponding electron diffraction and crystalline, indicating the arrangements of (200) and (110). (One representative data was shown from three independently repeated experiments)

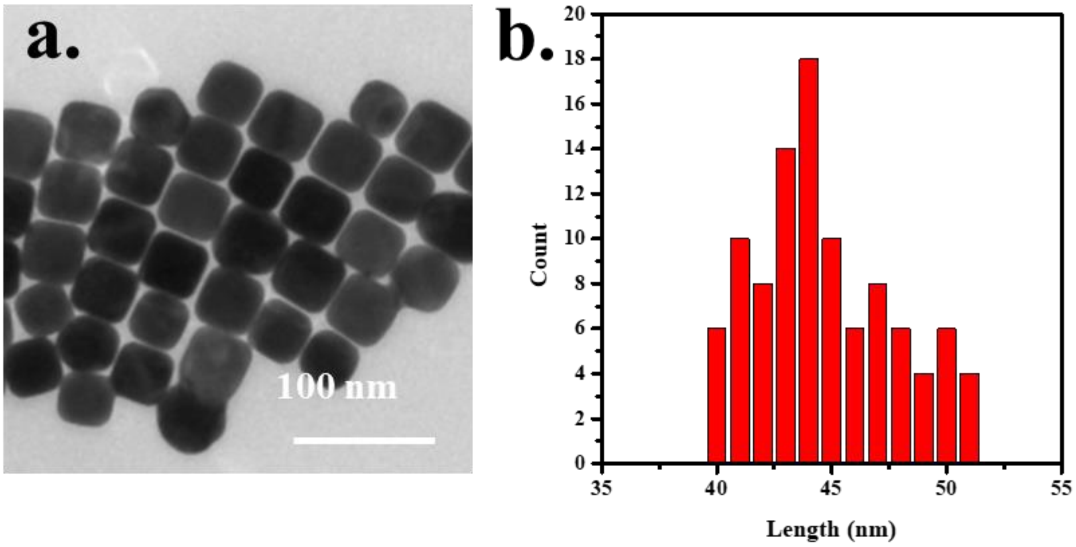

Supplementary Fig. 2. The characteristics of Au nanocubes. **a.** TEM image; **b.** Size distribution in edge length of Au nanocubes. (One representative data was shown from three independently repeated experiments)

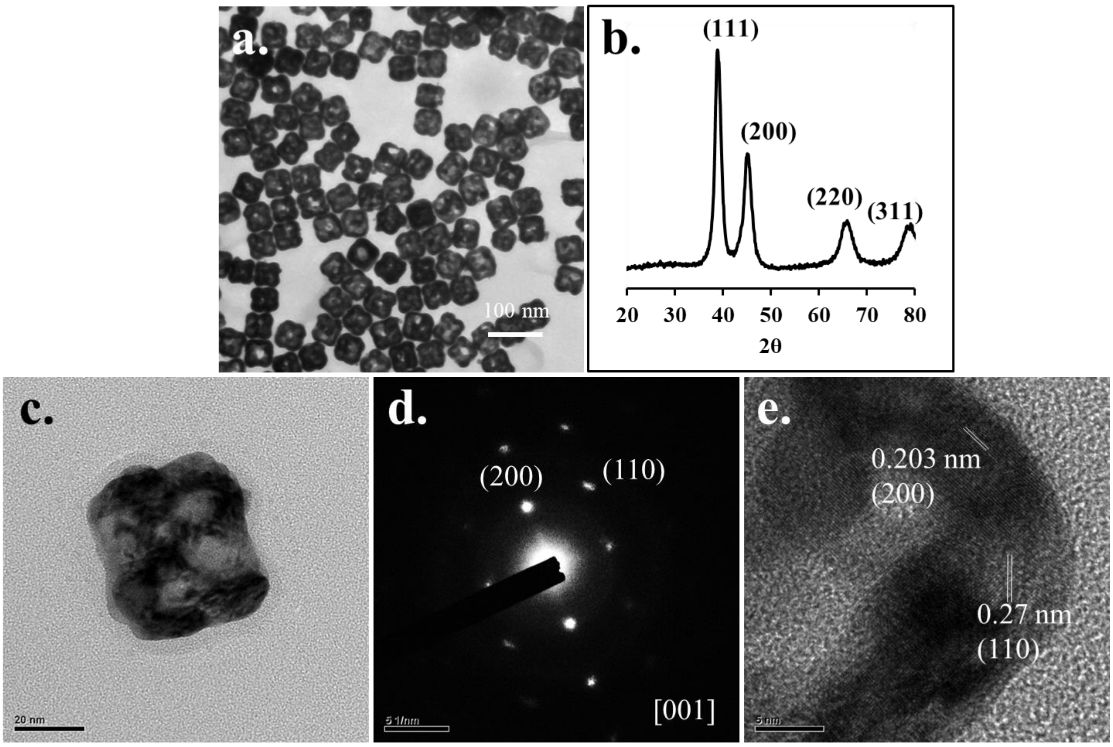

Supplementary Fig. 3. The characteristics of the alloyed $Au_{0.75}Cu_{0.25}$ nanocages through 60 μL $HAuCl_4$ (50 mM) addition. **a.** TEM image; **b.** XRD pattern of $Au_{0.75}Cu_{0.25}$ nanocages indicating the alloyed $Au_{0.75}Cu_{0.25}$ nanocage with a face-centered cubic crystal structure (JCPDS cards 01-071-5023); **c-e.** High-resolution TEM image of $Au_{0.75}Cu_{0.25}$ nanocage and its electron diffraction indicating the arrangements of (200) and (110). (One representative data was shown from three independently repeated experiments)

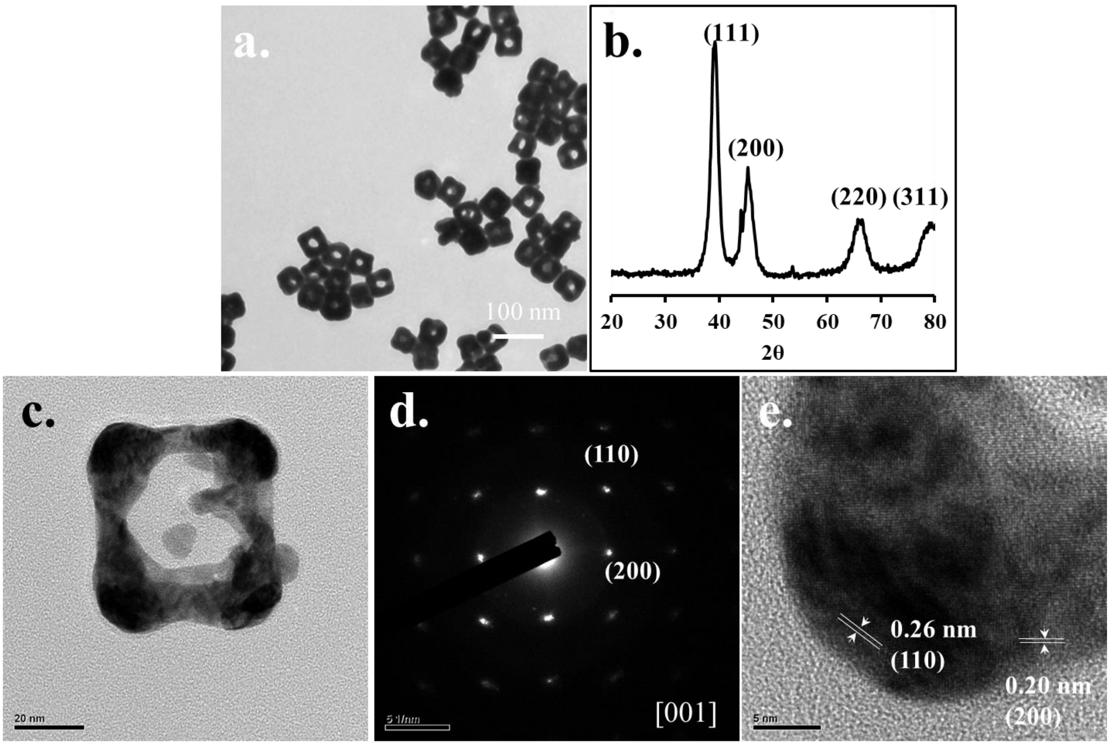

Supplementary Fig. 4. The characteristics of the alloyed $Au_{0.8}Cu_{0.2}$ nanoframes through 80 μL $HAuCl_4$ (50 mM) addition. **a.** TEM image; **b.** XRD pattern indicating the $Au_{0.8}Cu_{0.2}$ nanoframes with a face-centered cubic crystal structure (JCPDS cards 01-072-5241); **c-e.** High-resolution TEM image of $Au_{0.75}Cu_{0.25}$ nanoframe and its electron diffraction indicating the arrangements of (200) and (110). (One representative data was shown from three independently repeated experiments)

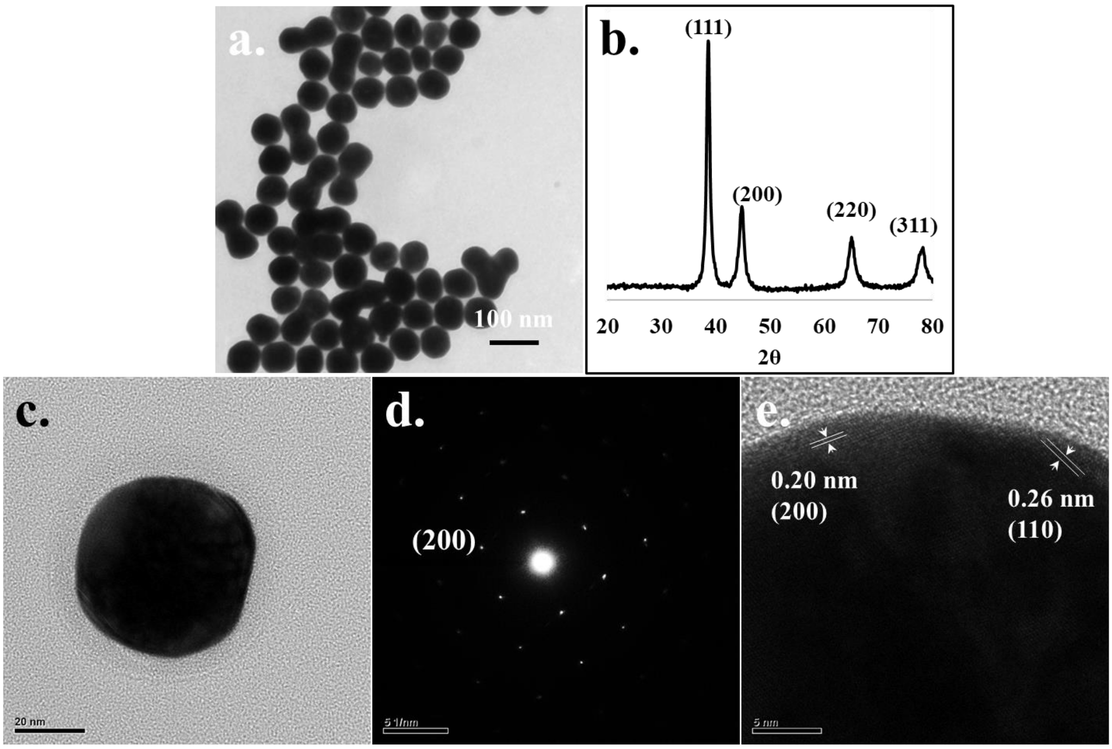

Supplementary Fig. 5. The characteristics of the completed replacement yielding Au nanoparticles through 100 μL $HAuCl_4$ (50 mM) addition. **a.** TEM image ; **b.** XRD pattern indicating the Au nanoparticles with a face-centered cubic crystal structure (JCPDS cards 04-0784); **c-e.** High-resolution TEM image of the Au nanoparticle and its electron diffraction indicating the arrangements of (200) and (110). (One representative data was shown from three independently repeated experiments)

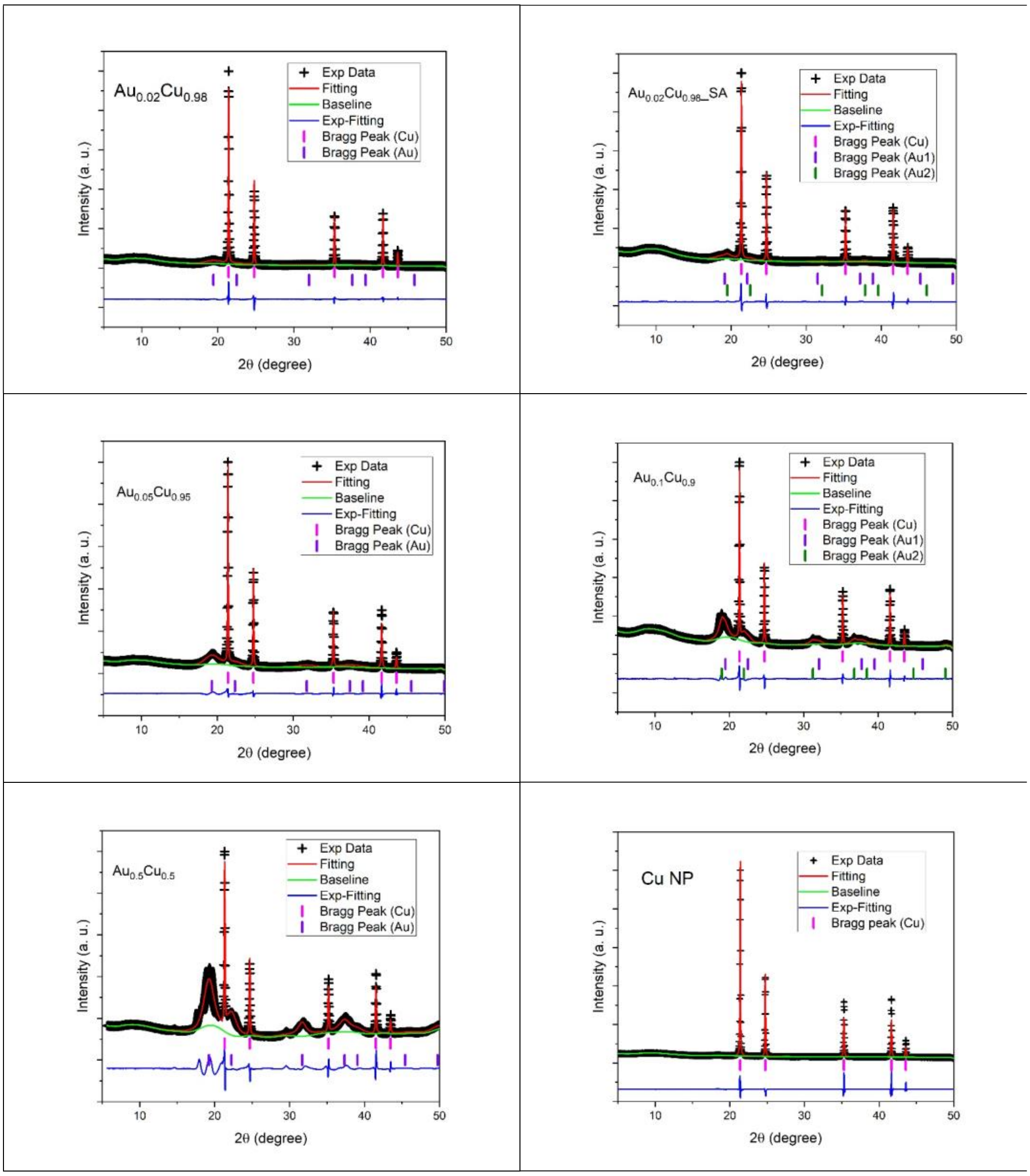

Supplementary Fig. 6. Rietveld refinement of Au/Cu^0 nanocubes. Powder diffraction data were collected using 16 keV (X-ray wavelength of 0.7749Å).

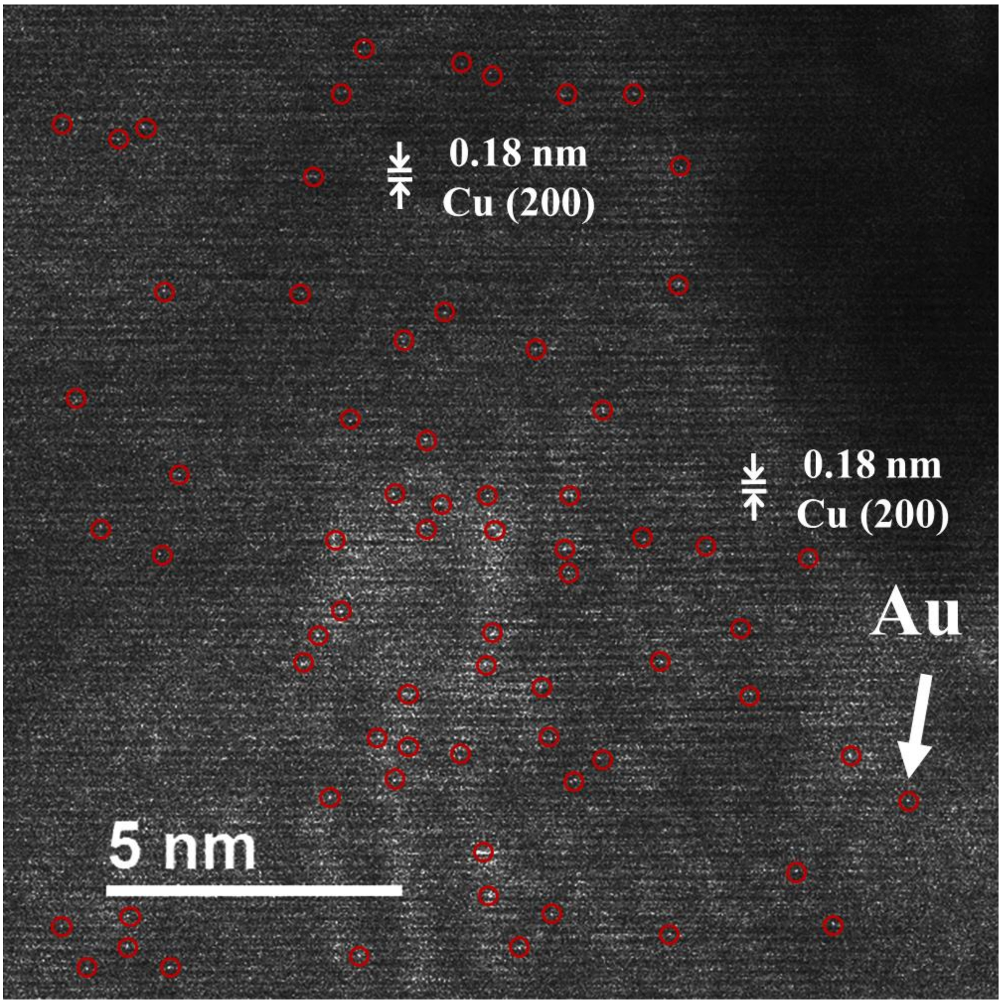

Supplementary Fig. 7. The magnified image of Fig. 1r showing high resolution of AC HAADF-STEM image of the $Au_{0.02}Cu_{0.98}$ nanocube. (One representative data was shown from three independently repeated experiments)

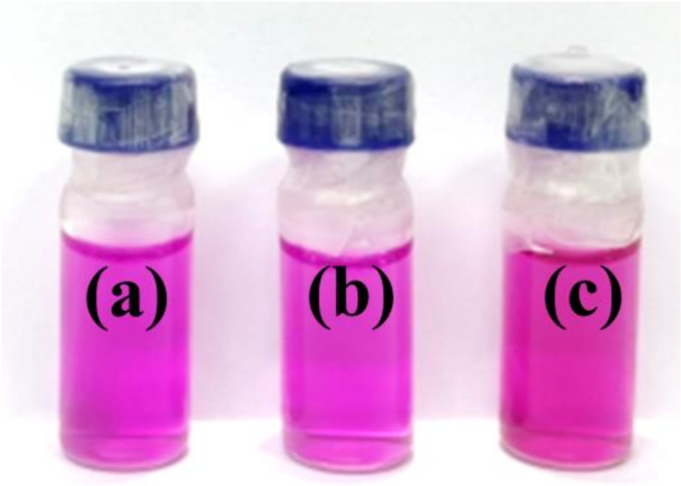

Supplementary Fig. 8. Examination of H_2O_2 generation in colloidal color by $KMnO_4$ with **(a)** blank, **(b)** $Au_{0.02}Cu_{0.98}$ (50 mg L^{-1}) and **(c)** $Au_{0.02}Cu_{0.98}$ (100 mg L^{-1}) under N_2 for 7 days.

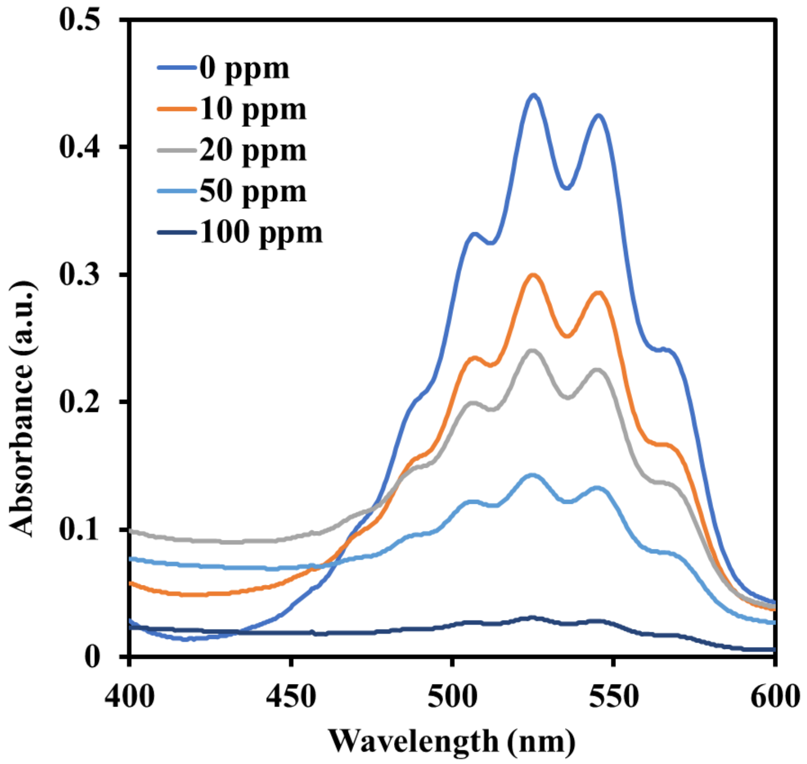

Supplementary Fig. 9. UV-Vis profiles in colorimetric analysis revealed the absorption intensity at 525 nm. The generation of H_2O_2 reduces MnO_4^- to colorless Mn^{2+} under different concentrations of $Au_{0.02}Cu_{0.98}$ nanocubes.

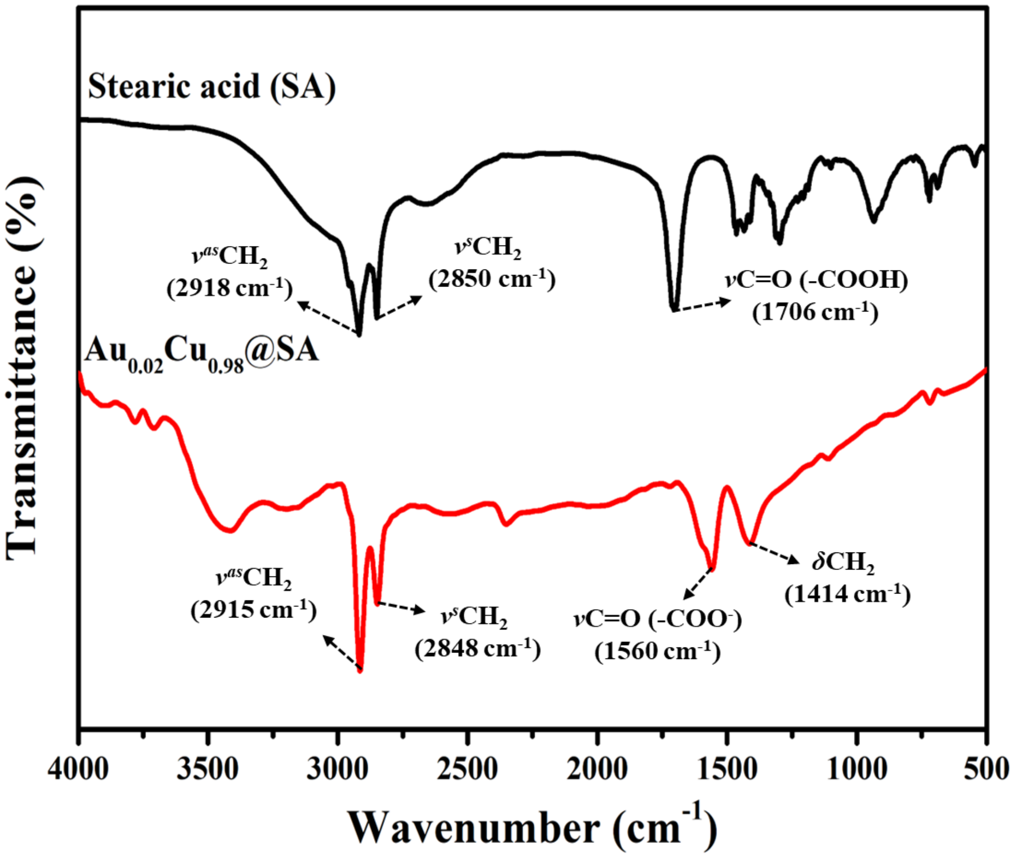

Supplementary Fig. 10. Fourier-transform infrared (FTIR) spectra of $Au_{0.02}Cu_{0.98}$ nanocubes with stearic acid (SA) modification. Two peaks at 2915 and 2848 cm^{-1} were respectively assigned as the asymmetric and symmetric stretching vibrations of CH in the CH_2 groups. The band at 1560 cm^{-1} was downshifted from 1706 cm^{-1} (SA), which was associated with the stretching frequencies of the C-O bonds of the −COOH groups after the surface modification on the $Au_{0.02}Cu_{0.98}$ nanocubes.

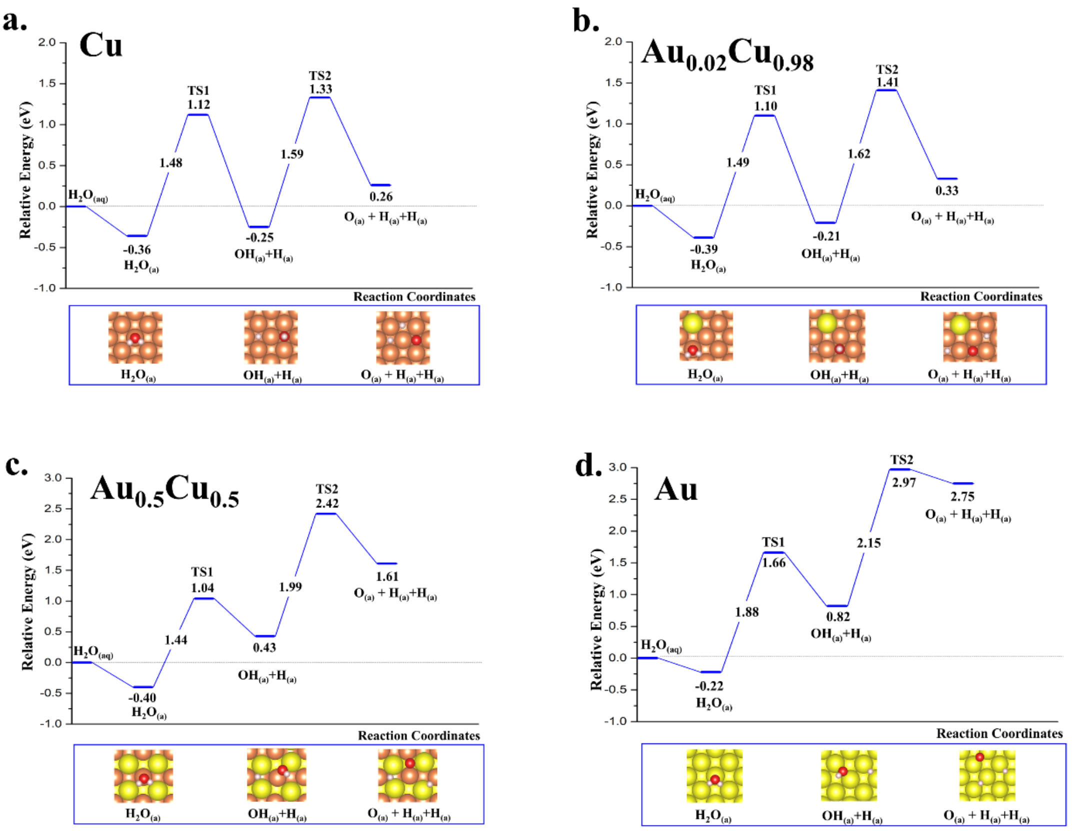

Supplementary Fig. 11. The calculated potential energy profiles of hydrogen production from H_2O dissociation on the **a.** pure Cu, **b.** $Au_{0.02}Cu_{0.98}$, **c.** $Au_{0.5}Cu_{0.5}$, and **d.** pure Au. Brown, gold, red and write spheres represent Cu, Au, O, and H atoms, respectively.

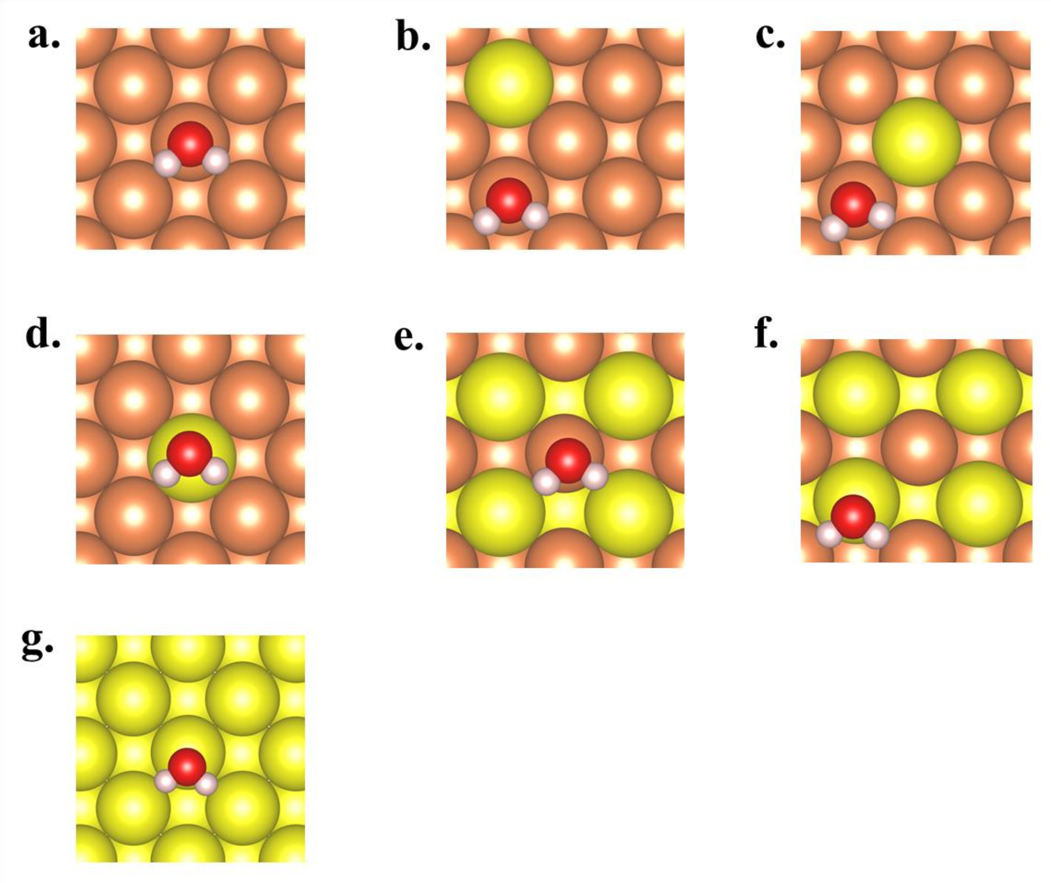

Supplementary Fig. 12. Optimized adsorption structures of H_2O molecule on **a.** T site at Cu(100) surface; **b.** T_1, **c.** T_2, and **d.** T_3 sites at $Au_{0.02}Cu_{0.98}(100)$ surface; **e.** T_1, and **f.** T_2 sites at $Au_{0.5}Cu_{0.5}(100)$ surface; **g.** T site at Au(100) surface. Brown, gold, red and write spheres represent Cu, Au, O, and H atoms, respectively.

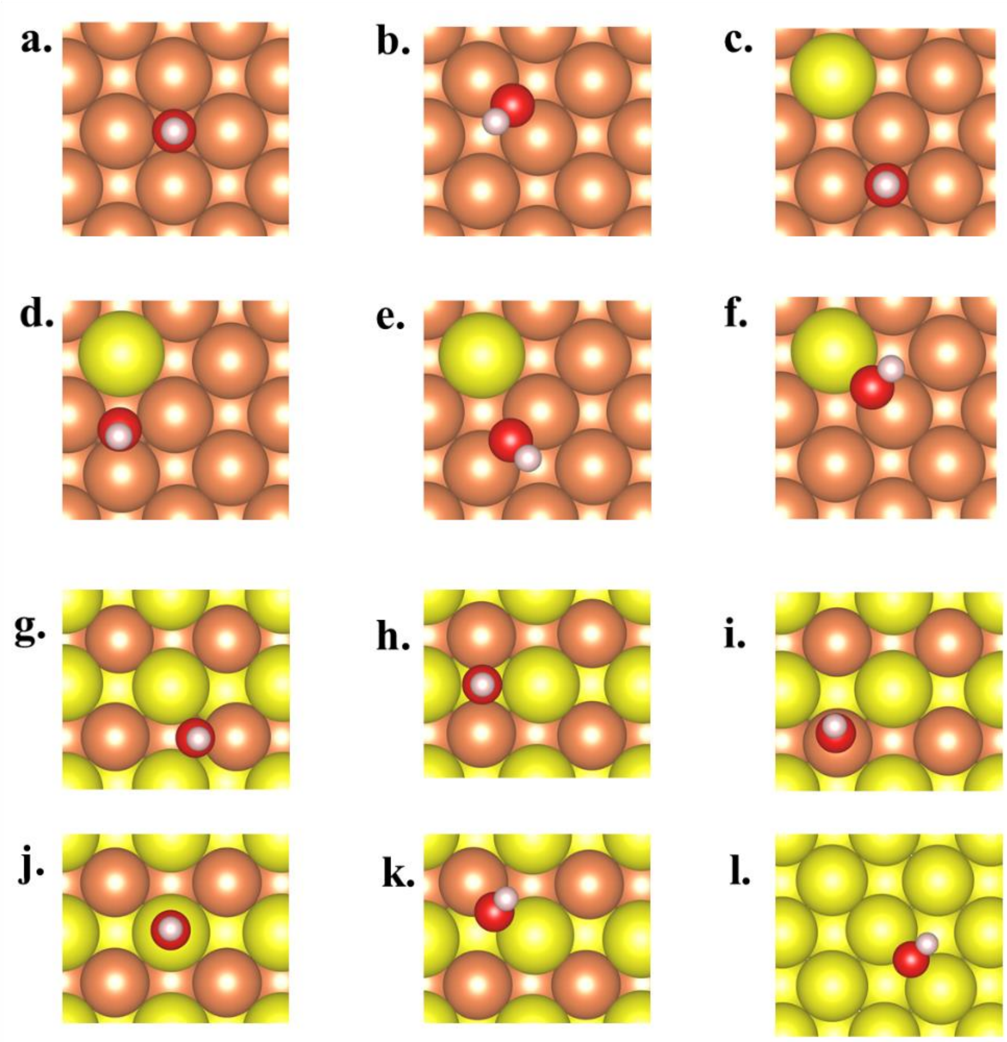

Supplementary Fig. 13. Optimized adsorption structures of OH group on **a.** H site, and **b.** B site at Cu(100) surface; **c.** H_1, **d.** H_2, **e**. B_1, and **f.** B_2 sites at $Au_{0.02}Cu_{0.98}(100)$ surface; **g**. H_1, **h**. H_2, **i.** T_1, **j.** T_2, and **k.** B sites at $Au_{0.5}Cu_{0.5}(100)$ surface; **l.** B site at Au(100) surface. Brown, gold, red and write spheres represent Cu, Au, O, and H atoms, respectively.

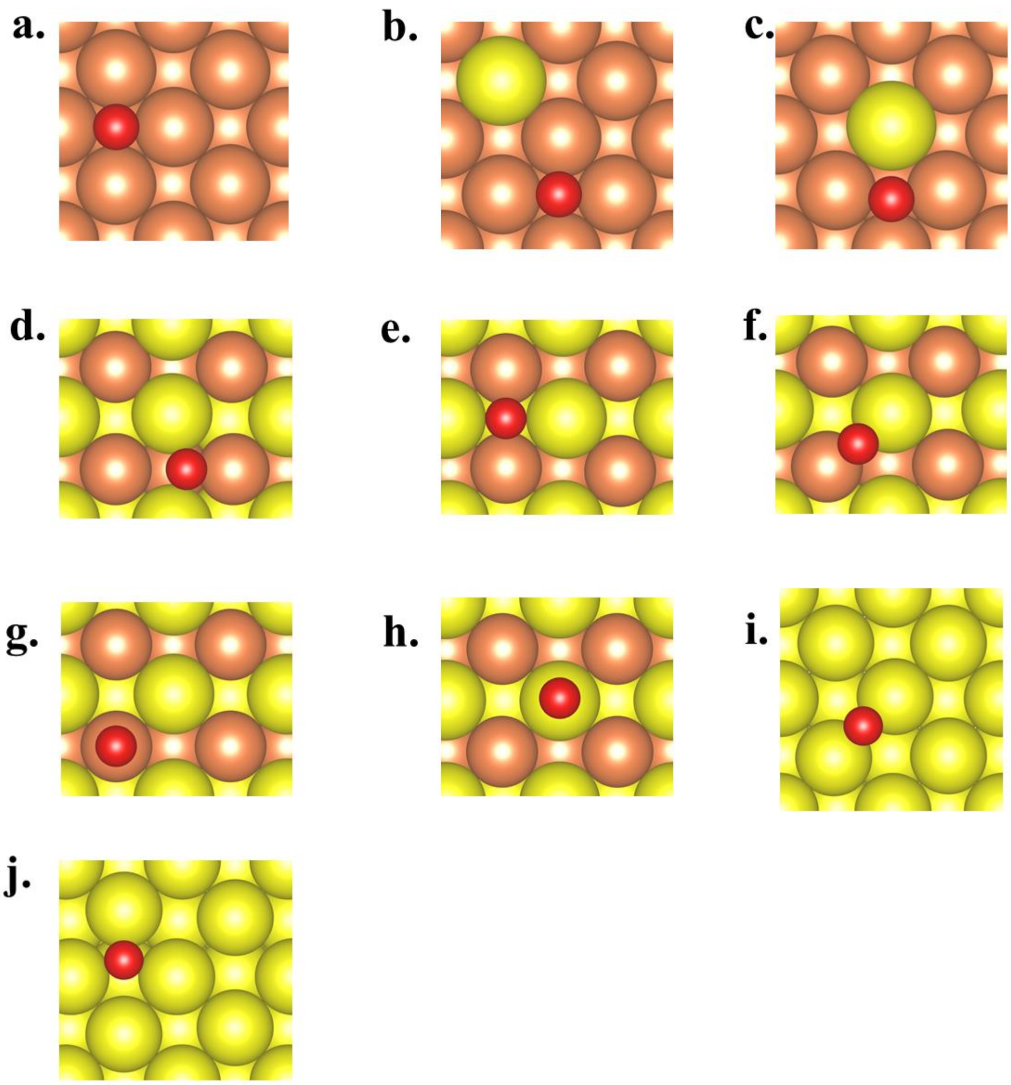

Supplementary Fig. 14. Optimized adsorption structures of O atom on **a.** H site at Cu(100) surface; **b.** H_1, and **c.** H_2 sites at $Au_{0.02}Cu_{0.98}$(100) surface; **d.** H_1, **e.** H_2, **f.** B, **g.** T_1, and **h.** T_2 sites at $Au_{0.5}Cu_{0.5}$(100) surface; **i.** B, and **j.** H site at Au(100) surface. Brown, gold, and red spheres represent Cu, Au, and O atoms, respectively.

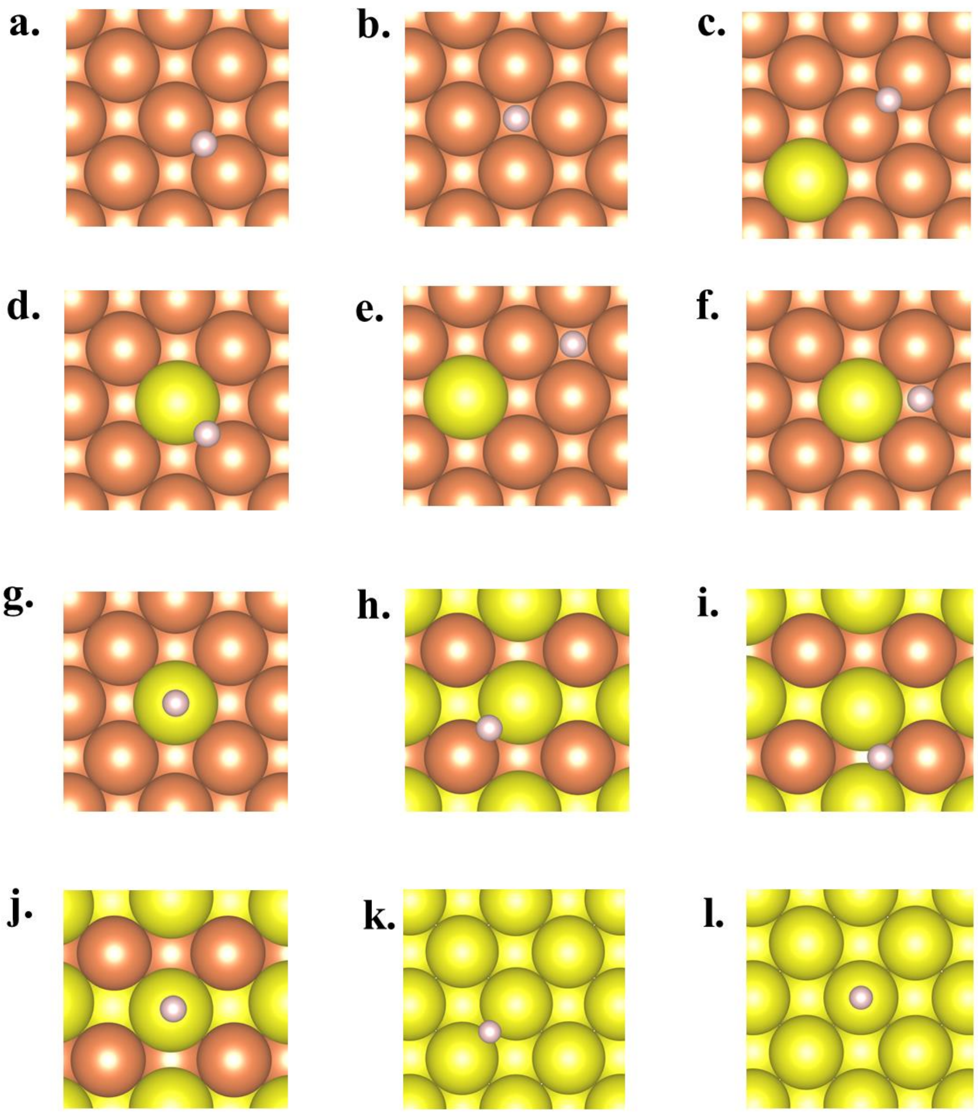

Supplementary Fig. 15. Optimized adsorption structures of H atom on **a.** B, and **b.** H sites at Cu(100) surface; **c.** B_1, **d.** B_2, **e.** H_1, **f.** H_2, and **g.** T_3 sites at $Au_{0.02}Cu_{0.98}(100)$ surface; **h.** B, **i.** H_1, and **j.** T_2 sites at $Au_{0.5}Cu_{0.5}(100)$ surface; **k.** B, and **l.** T sites at Au(100) surface. Brown, gold, and write spheres represent Cu, Au, and H atoms, respectively.

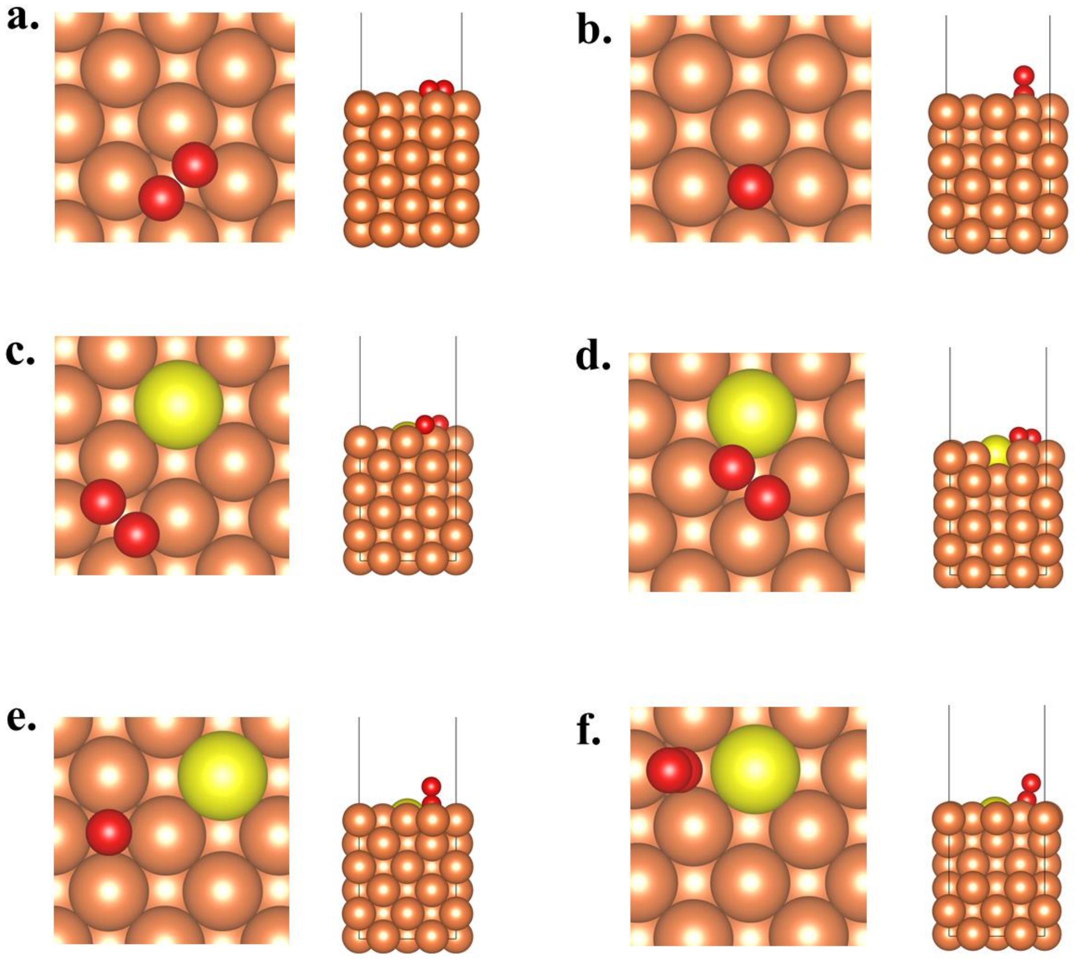

Supplementary Fig. 16. Optimized adsorption structures of O_2 molecule by different configurations on the metal surfaces: **a.** side-on, and **b.** end-on at H site on the Cu(100) surface; **c.** side-on at H_1 site, and **d.** side-on at H_2 site on the $Au_{0.02}Cu_{0.98}$(100) surface; **e.** end-on at H_1 site, and **f.** end-on at H_2 site on the $Au_{0.02}Cu_{0.98}$(100) surface. Brown, gold, and red spheres represent Cu, Au, and O atoms, respectively.

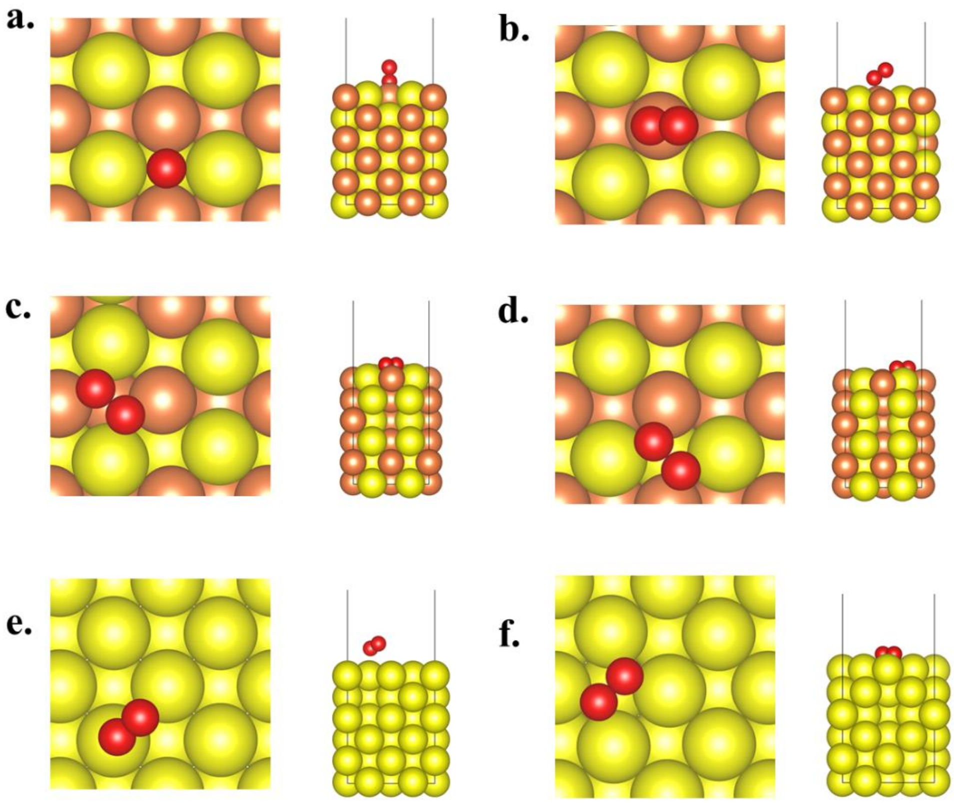

Supplementary Fig. 17. Optimized adsorption structures of O_2 molecule by different configurations on the metal surfaces: **a.** end-on at H_1 site, **b.** end-on at T_1 site, **c.** side-on at H_1 site, and **d.** side-on at H_2 site on the $Au_{0.5}Cu_{0.5}$(100) surface; **e.** end-on at T site, and **f.** side-on at H site on the Au(100) surface. Brown, gold, and red spheres represent Cu, Au, and O atoms, respectively.

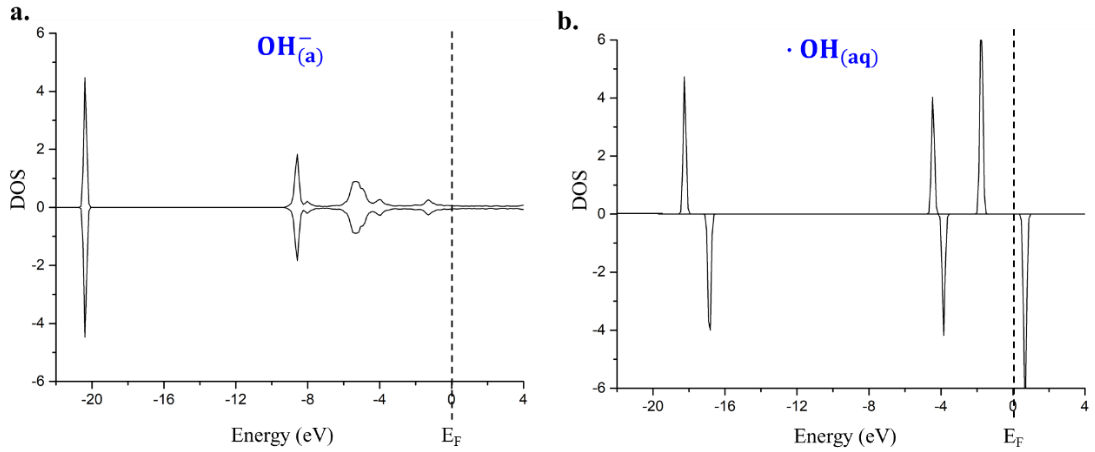

Supplementary Fig. 18. Calculated projected density of states diagrams of **a.** $OH^-_{(a)}$, and **b.** $\cdot OH_{(aq)}$ on the $Au_{0.02}Cu_{0.98}(100)$ surface. The dashed line represents the Fermi-level.

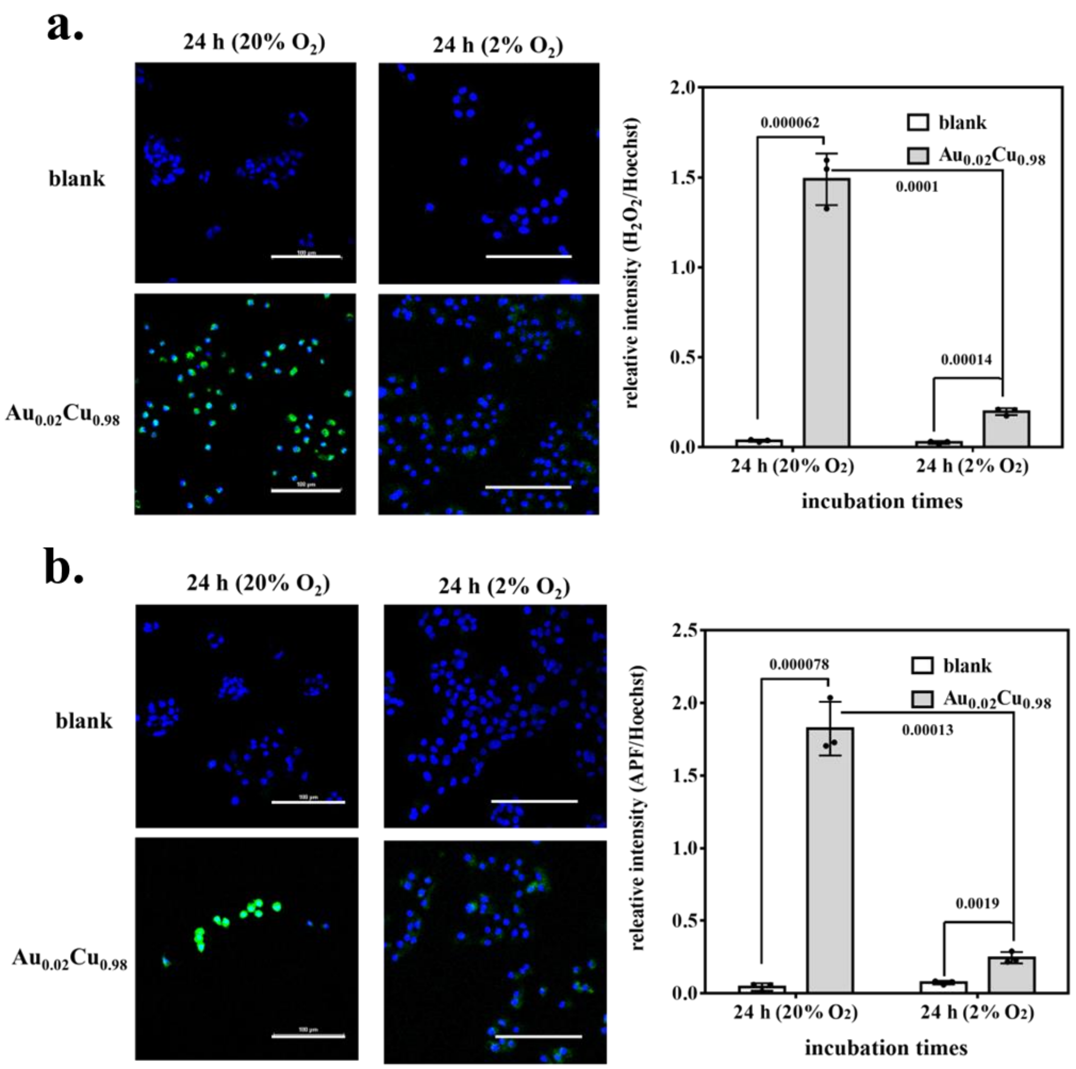

Supplementary Fig. 19. The quantitative analysis of **a.** H_2O_2, and **b.** •OH generation determined by respective hydrogen peroxide assay kit and APF dye under different O_2 concentrations at 2 % and 20 %. All data were obtained in triplicate (data were mean ± S.D. the p-values calculated by one-way ANOVA. Source data are provided as a Source Data file.).

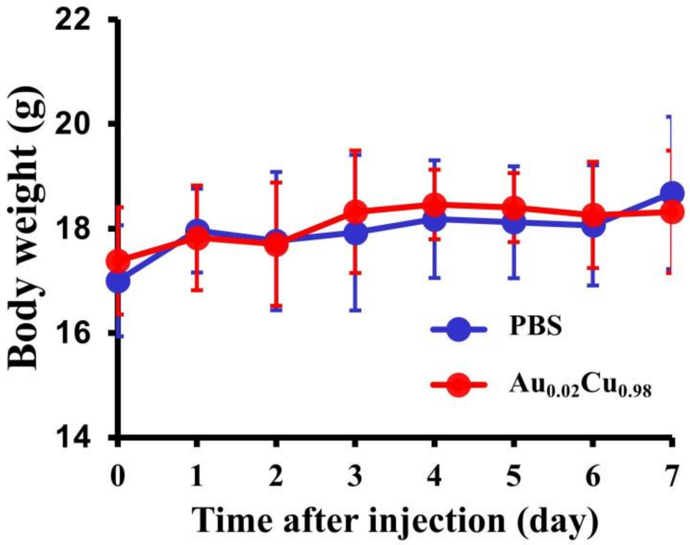

Supplementary Fig. 20. The body weight of C57BL/6 mice treated with PBS and $Au_{0.02}Cu_{0.98}$@SA nanocubes during 7 days post-treatment (n=5). The error bars in represented mean ± SEM.

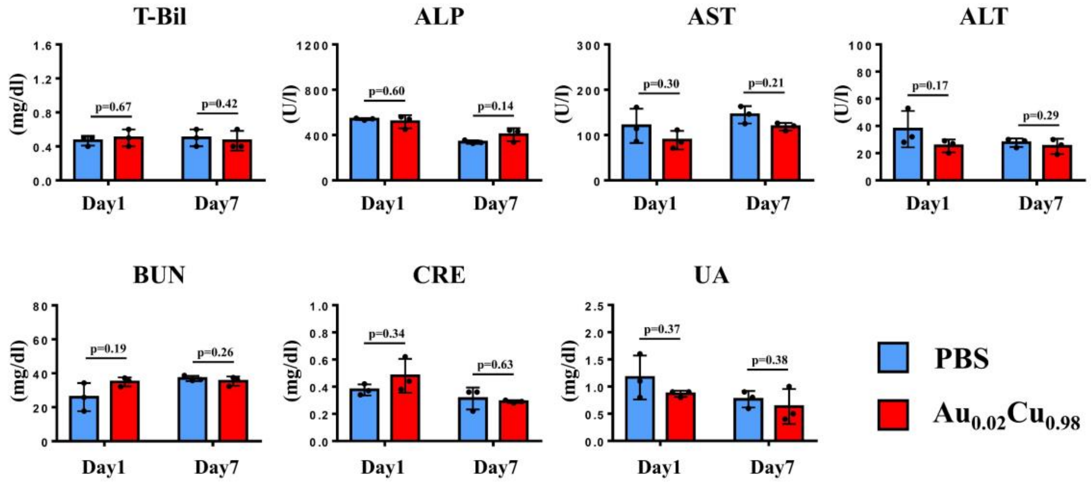

Supplementary Fig. S21. The blood biochemical analysis was determined on post-injection day 7 of C57BL/6 mice with PBS and $Au_{0.02}Cu_{0.98}$@SA nanocubes through intravenous injection (T-Bil: total bilirubin, ALP: alkaline phosphatase, AST: aspartate transaminase, ALT: alanine transaminase, BUN: blood urea nitrogen, CREA: creatinine, UA: uric acid, n=3). The error bars in represented mean ± SEM. The p-value was calculated by two-tailed Student's *t* test.

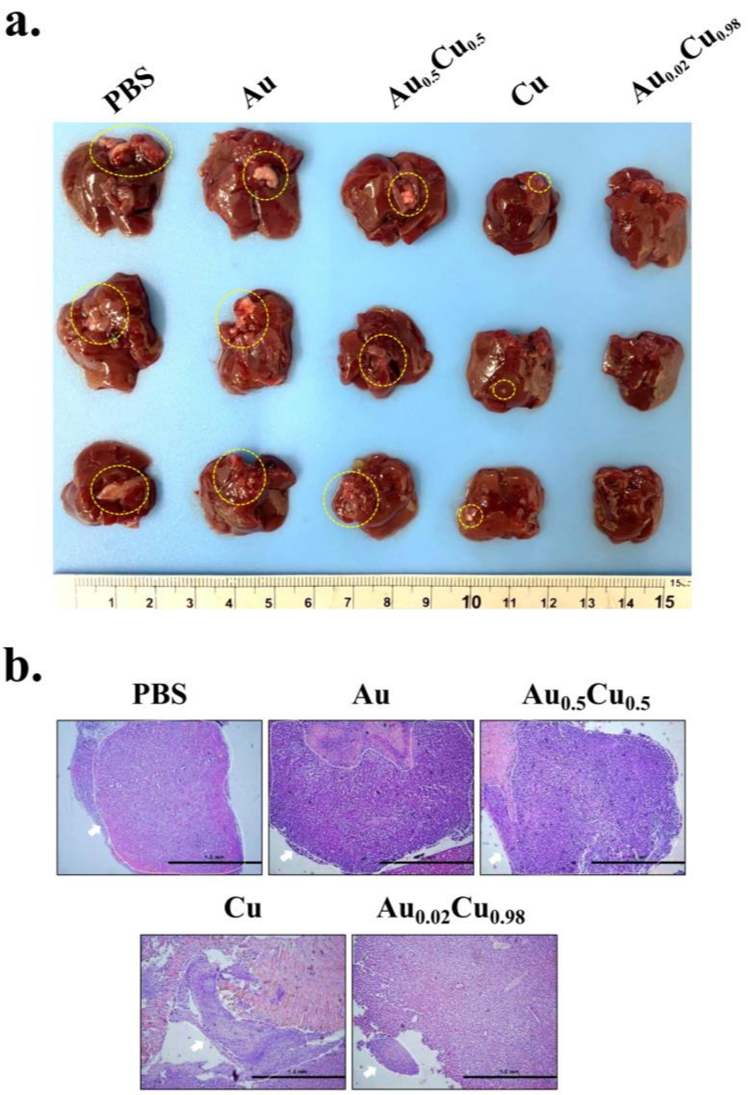

Supplementary Fig. S22. The histological morphology and area of hepatocellular carcinoma. a. The appearance of livers with hepatocellular carcinoma in each treatment group after mice sacrificed (n=3). The yellow circle indicated the tumor area. **b.** The morphology of hepatocellular carcinoma in each treated mice was examined by H&E staining (Scale bar, 1 mm). The enclosed white dot regions indicate the live tumor area.ed white dot regions indicate the live tumor area. The experiment of H&E staining was repeated at least three times independently with a similar tendency and the result from a representative experiment was shown.

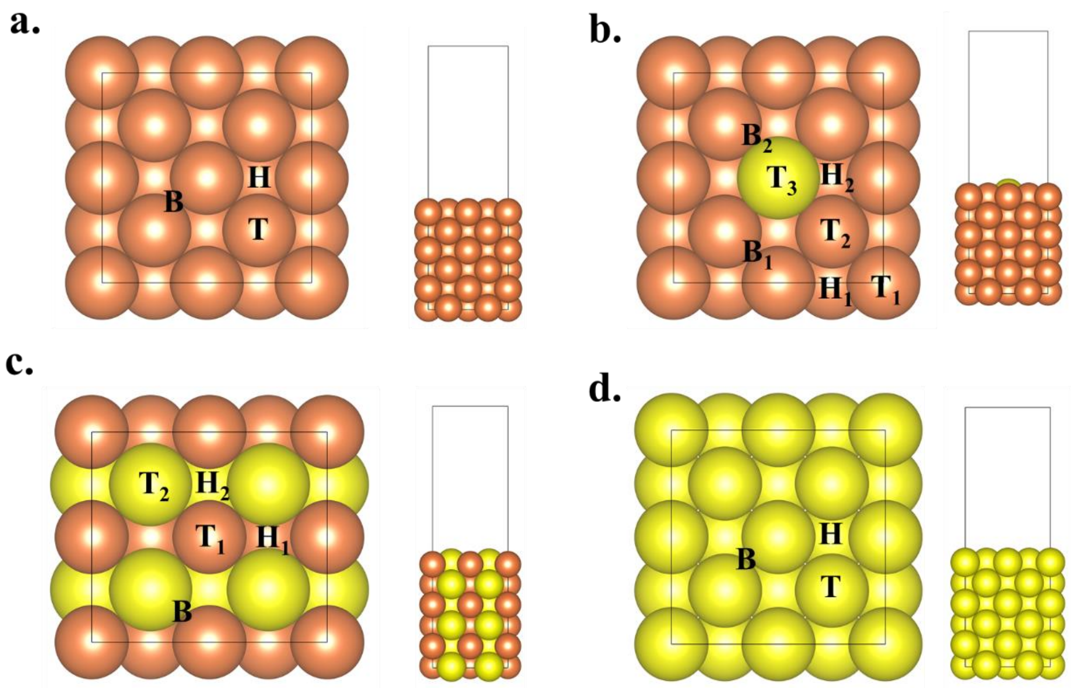

Supplementary Fig. S23. Top and side views of **a.** Cu(100), **b.** $Au_{0.02}Cu_{0.98}$(100), **c.** $Au_{0.5}Cu_{0.5}$(100), and **d**. Au(100) surfaces, where the T, B, and H represent the top, bridge, and hollow sites. Brown and gold spheres represent Cu and Au atoms, respectively.

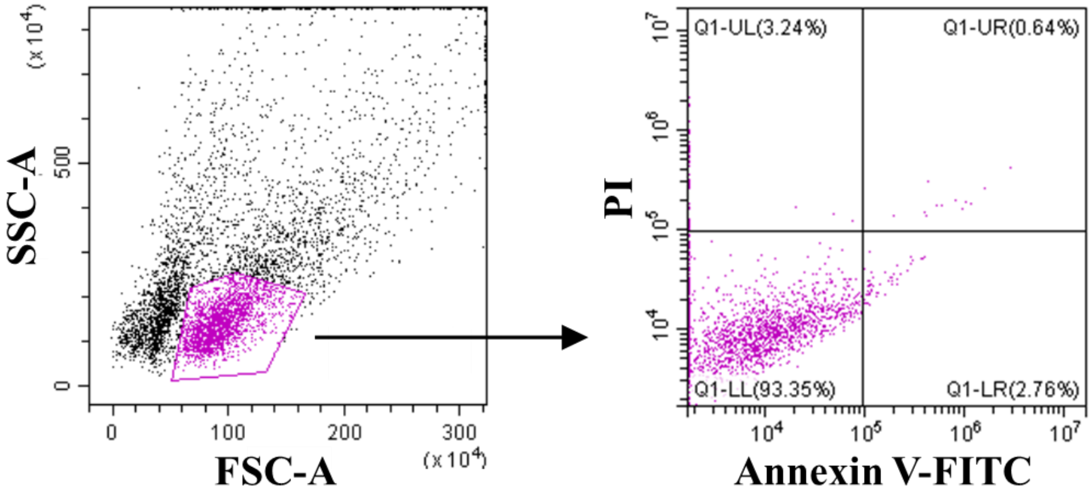

Supplementary Fig. S24. Figure exemplifying the gating strategy for HepG2-Red-FLuc hepatocellular carcinoma cells for flow cytometry analysis in Fig. 5d.
